# 3-Bromo­pyridin-2-amine

**DOI:** 10.1107/S1600536811055541

**Published:** 2012-01-14

**Authors:** Marcelle Johnson, Andreas Lemmerer

**Affiliations:** a Molecular Sciences Institute, School of Chemistry, University of the Witwatersrand, Johannesburg, PO Wits 2050, South Africa

## Abstract

In the crystal structure of the title compound, C_5_H_5_BrN_2_, mol­ecules assemble *via* pairs of N—H⋯N hydrogen bonds into inversion dimers using only the *syn* H atom on the amine group. These dimers then assemble further into two-dimensional layers *via* type I C—Br⋯Br [Br⋯Br = 3.693 (s6) Å] halogen bonding along the (102) plane.

## Related literature

For halogen bonding, see: Metrangelo *et al.* (2005 ▶). For a related structure, see: Hu *et al.* (2011 ▶).
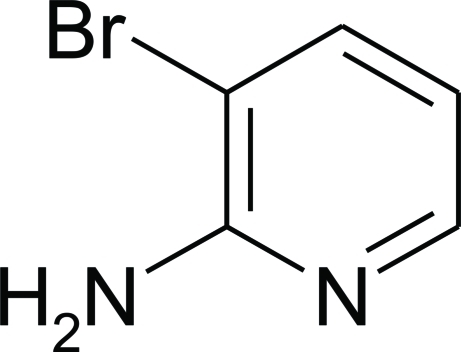


## Experimental

### 

#### Crystal data

C_5_H_5_BrN_2_
*M* *_r_* = 173.02
Monoclinic, 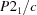

*a* = 12.2179 (6) Å
*b* = 4.0007 (2) Å
*c* = 12.8451 (6) Å
β = 109.731 (3)°
*V* = 591.01 (5) Å^3^
*Z* = 4
Mo *K*α radiation
μ = 6.84 mm^−1^
*T* = 173 K
0.5 × 0.4 × 0.09 mm

#### Data collection

Bruker SMART APEXII CCD area-detector diffractometer
Absorption correction: integration (*XPREP*; Bruker, 2004 ▶) *T*_min_ = 0.131, *T*_max_ = 0.578
5622 measured reflections
1428 independent reflections
1200 reflections with *I* > 2σ(*I*)
*R*_int_ = 0.093

#### Refinement

*R*[*F* ^2^ > 2σ(*F* ^2^)] = 0.034
*wR*(*F* ^2^) = 0.082
*S* = 0.99
1428 reflections
81 parameters
H atoms treated by a mixture of independent and constrained refinement
Δρ_max_ = 1.04 e Å^−3^
Δρ_min_ = −0.77 e Å^−3^

### 

Data collection: *APEX2* (Bruker, 2005 ▶); cell refinement: *SAINT-Plus* (Bruker, 2004 ▶); data reduction: *SAINT-Plus* and *XPREP* (Bruker 2004 ▶); program(s) used to solve structure: *SHELXS97* (Sheldrick, 2008 ▶); program(s) used to refine structure: *SHELXL97* (Sheldrick, 2008 ▶); molecular graphics: *ORTEP-3 for Windows* (Farrugia, 1997 ▶) and *DIAMOND* (Brandenburg, 1999 ▶); software used to prepare material for publication: *WinGX* (Farrugia, 1999 ▶) and *PLATON* (Spek, 2009 ▶).

## Supplementary Material

Crystal structure: contains datablock(s) global, I. DOI: 10.1107/S1600536811055541/zj2050sup1.cif

Supplementary material file. DOI: 10.1107/S1600536811055541/zj2050Isup2.mol

Structure factors: contains datablock(s) I. DOI: 10.1107/S1600536811055541/zj2050Isup3.hkl

Supplementary material file. DOI: 10.1107/S1600536811055541/zj2050Isup4.cml

Additional supplementary materials:  crystallographic information; 3D view; checkCIF report

## Figures and Tables

**Table 1:** Hydrogen-bond geometry (Å, °)

*D*—H⋯*A*	*D*—H	H⋯*A*	*D*⋯*A*	*D*—H⋯*A*
N2—H2*S*⋯N1^i^	0.81 (4)	2.21 (4)	3.019 (4)	173 (3)

Symmetry code: (i) 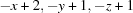.

**Fig. 1.**
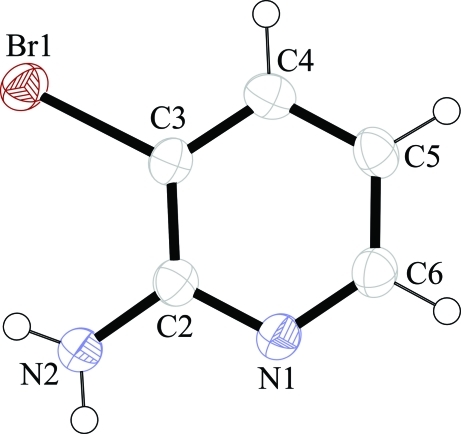
The asymmetric unit of (I) showing the atomic numbering scheme. Displacement ellipsoids are shown at the 50% probability level.

**Fig. 2.**
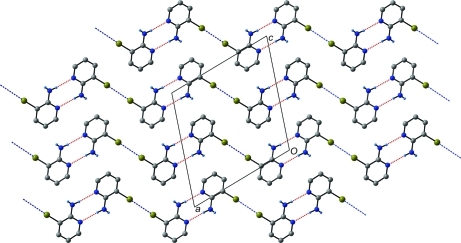
Packing diagram of (I). Intermolecular N—H···N hydrogen bonds are shown as dashed red lines forming dimers. Note that the anti H is not used in any hydrogen bonding interactions. The C—Br···Br halogen bonds are shown as dashed blue lines.

## supplementary crystallographic information

### Comment

The title compound is being used as a co-crystal former for potential co-crystal with the molecule 2-chloro-4-nitrobenzoic acid. As its structure has not been determined previously, and for screening purposes, it is now reported (Fig. 1). The title molecule forms centrosymmetric dimers using the *syn* H2S atom on the amine group. The *anti* H atom H2A is not involved in any intermolecular interactions. The dimers are joined by type II C—Br···Br halogen bonding (Metrangelo *et al.*, 2005) to form 2-D layers (Fig. 2). The related compound, 3-chloropyridin-2-amine (Hu *et al.*, 2011), has the same hydrogen bonded dimers, but forms instead chains of dimers through C—Cl···Cl halogen bonding of type I.

### Experimental

Crystals were grown by slow evaporation of a methanol solution of the title compound, 0.200 g (1.16 mmol) in 8 ml of methanol, and afforded light brown plates after three days of slow evaporation at ambient conditions.

### Refinement

The aromatic C-bound H atoms were geometrically placed, C—H bond length of 0.95 Å and refined as riding with *U*_iso_(H) = 1.2*U*_eq_(C). The N-bound H atoms were located in the difference map and coordinates as well as isotropic displacement parameters refined freely.

### Crystal data

**Table:**

C_5_H_5_BrN_2_	*F*(000) = 336
*M**_r_* = 173.02	*D*_x_ = 1.945 Mg m^−^^3^
Monoclinic, *P*2_1_/*c*	Mo *K*α radiation, λ = 0.71073 Å
Hall symbol: -P 2ybc	Cell parameters from 2888 reflections
*a* = 12.2179 (6) Å	θ = 3.2–28.3°
*b* = 4.0007 (2) Å	µ = 6.84 mm^−^^1^
*c* = 12.8451 (6) Å	*T* = 173 K
β = 109.731 (3)°	Plate, brown
*V* = 591.01 (5) Å^3^	0.5 × 0.4 × 0.09 mm
*Z* = 4	

### Data collection

**Table:**

Bruker SMART APEXII CCD area-detector diffractometer	1200 reflections with *I* > 2σ(*I*)
ω scans	*R*_int_ = 0.093
Absorption correction: integration (*XPREP*; Bruker, 2004)	θ_max_ = 28.0°, θ_min_ = 1.8°
*T*_min_ = 0.131, *T*_max_ = 0.578	*h* = −16→15
5622 measured reflections	*k* = −5→5
1428 independent reflections	*l* = −16→16

### Refinement

**Table:**

Refinement on *F*^2^	0 restraints
Least-squares matrix: full	H atoms treated by a mixture of independent and constrained refinement
*R*[*F*^2^ > 2σ(*F*^2^)] = 0.034	*w* = 1/[σ^2^(*F*_o_^2^) + (0.0479*P*)^2^] where *P* = (*F*_o_^2^ + 2*F*_c_^2^)/3
*wR*(*F*^2^) = 0.082	(Δ/σ)_max_ < 0.001
*S* = 0.99	Δρ_max_ = 1.04 e Å^−^^3^
1428 reflections	Δρ_min_ = −0.77 e Å^−^^3^
81 parameters	

### Special details

**Table:**

Experimental. Numerical integration absorption corrections based on indexed crystal faces were applied using the *XPREP* routine (Bruker, 2004)
Geometry. All e.s.d.'s (except the e.s.d. in the dihedral angle between two l.s. planes) are estimated using the full covariance matrix. The cell e.s.d.'s are taken into account individually in the estimation of e.s.d.'s in distances, angles and torsion angles; correlations between e.s.d.'s in cell parameters are only used when they are defined by crystal symmetry. An approximate (isotropic) treatment of cell e.s.d.'s is used for estimating e.s.d.'s involving l.s. planes.

### Fractional atomic coordinates and isotropic or equivalent isotropic displacement parameters (Å^2^)

**Table:**

	*x*	*y*	*z*	*U*_iso_*/*U*_eq_	
C2	0.8182 (2)	0.4235 (7)	0.4926 (2)	0.0288 (6)	
C3	0.7048 (2)	0.3668 (6)	0.4912 (2)	0.0267 (5)	
C4	0.6702 (2)	0.4704 (7)	0.5771 (2)	0.0309 (6)	
H4	0.5934	0.4287	0.5763	0.037*	
C5	0.7499 (3)	0.6374 (7)	0.6653 (2)	0.0327 (6)	
H5	0.729	0.7168	0.7258	0.039*	
C6	0.8602 (3)	0.6833 (7)	0.6615 (2)	0.0334 (6)	
H6	0.915	0.7976	0.7215	0.04*	
N1	0.8956 (2)	0.5781 (6)	0.5793 (2)	0.0325 (5)	
N2	0.8547 (3)	0.3359 (7)	0.4077 (2)	0.0386 (6)	
Br1	0.59557 (2)	0.15619 (6)	0.36599 (2)	0.03201 (13)	
H2S	0.923 (3)	0.345 (7)	0.415 (3)	0.030 (9)*	
H2A	0.820 (4)	0.210 (8)	0.362 (4)	0.043 (11)*	

### Atomic displacement parameters (Å^2^)

**Table:**

	*U*^11^	*U*^22^	*U*^33^	*U*^12^	*U*^13^	*U*^23^
C2	0.0309 (14)	0.0294 (12)	0.0253 (12)	0.0002 (11)	0.0084 (11)	0.0031 (10)
C3	0.0271 (14)	0.0256 (12)	0.0238 (12)	0.0031 (9)	0.0039 (11)	0.0034 (9)
C4	0.0282 (14)	0.0331 (13)	0.0319 (13)	0.0070 (11)	0.0107 (11)	0.0076 (12)
C5	0.0359 (16)	0.0376 (15)	0.0250 (13)	0.0059 (11)	0.0109 (12)	0.0036 (10)
C6	0.0343 (16)	0.0367 (15)	0.0273 (13)	−0.0010 (12)	0.0079 (12)	0.0002 (11)
N1	0.0286 (13)	0.0408 (12)	0.0266 (11)	−0.0041 (10)	0.0073 (10)	−0.0022 (10)
N2	0.0303 (15)	0.0568 (18)	0.0309 (13)	−0.0112 (12)	0.0134 (12)	−0.0120 (12)
Br1	0.02548 (18)	0.03463 (19)	0.03161 (18)	0.00029 (10)	0.00396 (12)	−0.00178 (10)

### Geometric parameters (Å, °)

**Table:**

C2—N1	1.344 (4)	C5—C6	1.378 (5)
C2—N2	1.357 (4)	C5—H5	0.95
C2—C3	1.398 (4)	C6—N1	1.336 (4)
C3—C4	1.372 (4)	C6—H6	0.95
C3—Br1	1.904 (3)	N2—H2S	0.81 (4)
C4—C5	1.390 (4)	N2—H2A	0.78 (4)
C4—H4	0.95		
			
N1—C2—N2	117.1 (3)	C6—C5—H5	121.3
N1—C2—C3	120.2 (2)	C4—C5—H5	121.3
N2—C2—C3	122.7 (3)	N1—C6—C5	124.6 (3)
C4—C3—C2	120.8 (3)	N1—C6—H6	117.7
C4—C3—Br1	119.7 (2)	C5—C6—H6	117.7
C2—C3—Br1	119.5 (2)	C6—N1—C2	118.4 (2)
C3—C4—C5	118.7 (3)	C2—N2—H2S	120 (3)
C3—C4—H4	120.6	C2—N2—H2A	122 (3)
C5—C4—H4	120.6	H2S—N2—H2A	113 (4)
C6—C5—C4	117.3 (3)		
			
N1—C2—C3—C4	0.7 (4)	C3—C4—C5—C6	−1.1 (4)
N2—C2—C3—C4	−177.6 (3)	C4—C5—C6—N1	−0.1 (4)
N1—C2—C3—Br1	178.6 (2)	C5—C6—N1—C2	1.7 (4)
N2—C2—C3—Br1	0.3 (4)	N2—C2—N1—C6	176.5 (3)
C2—C3—C4—C5	0.9 (4)	C3—C2—N1—C6	−1.9 (4)
Br1—C3—C4—C5	−177.04 (19)		

### Hydrogen-bond geometry (Å, °)

**Table:**

*D*—H···*A*	*D*—H	H···*A*	*D*···*A*	*D*—H···*A*
N2—H2S···N1^i^	0.81 (4)	2.21 (4)	3.019 (4)	173 (3)

Symmetry codes: (i) −*x*+2, −*y*+1, −*z*+1.
